# Optimal Conversion of Food Packaging Waste to Liquid Fuel via Nonthermal Plasma Treatment: A Model-Centric Approach

**DOI:** 10.3390/polym16212990

**Published:** 2024-10-25

**Authors:** Mohammad Jakir Hossain Khan, Zilvinas Kryzevicius, Audrius Senulis, Audrone Zukauskaite, Paulius Rapalis, Jochen Uebe

**Affiliations:** 1 Engineering Department, Faculty of Marine Technology and Natural Sciences, Klaipeda University, H. Manto 84, 92294 Klaipeda, Lithuania; khan.mohammad-jakir-hossain@ku.lt (M.J.H.K.); zilvinas.kryzevicius@ku.lt (Z.K.); audrius.senulis@ku.lt (A.S.); audrone.zukauskaite@ku.lt (A.Z.); 2 Marine Research Institute, Klaipeda University, H. Manto 84, 92294 Klaipeda, Lithuania; paulius.rapalis@ku.lt

**Keywords:** food packaging wastes, petroleum hydrocarbons, non-thermal plasma characteristics and optimisation

## Abstract

The efficiency of employing a multifactorial approach to enhance the nonthermal plasma (NTP) chemical conversion of solid waste food packaging materials into liquid petroleum hydrocarbons was assessed for the first time in this study. The researchers adopted a hybrid approach which integrated the zero-dimensional (0-D) and response surface model (RSM) techniques. After their application, the researchers noted that these strategies significantly enhanced the model prediction owing to their accurate electrochemical description. Here, the researchers solved a set of equations to identify the optimisation dynamics. They also established experimental circumstances to determine the quantitative correlation among all process variables contributing to food plastic packaging waste degradation and the production of liquid fuels. The findings of the study indicate a good agreement between the numerical and experimental values. It was also noted that the electrical variables of NTP significantly influenced the conversion yield (Y_conv%_) of solid plastic packaging waste to liquid hydrocarbons. Similarly, after analysing the data, it was seen that factors like the power discharge rate ($x_{1}$ ), discharge interval ($x_{2}$), power frequency ($x_{3}$), and power intensity ($x_{4}$) could significantly affect the product yield. After optimizing the variables, the researchers observed a maximal Y_conv%_ of approximately 86%. The findings revealed that the proposed framework could effectively scale up the plasma synergistic pyrolysis technology for obtaining the highest Y_conv%_ of solid packaging plastic wastes to produce an aromatics-enriched oil. The researchers subsequently employed the precision of the constructed framework to upgrade the laboratory-scale procedures to industrial-scale processes, which showed more than 95% efficiency. The extracted oil showed a calorific value of 43,570.5 J/g, indicating that the liquid hydrocarbons exhibited properties similar to commercial diesel.

## 1. Introduction

The development of the global middle class, along with an increase in trade and consumption, has resulted in a major increase in the use of plastic materials in food packaging industries [1]. However, the widespread usage of plastic packages presents additional environmental problems, like increased use of fossil and energy resources, as well as waste management problems like marine litter. Although plastics are effective and lightweight, their widespread usage as food packages and short lifespan results in a large quantity of post-use plastic packages [2]. Plastic packages play an important role in the transition to sustainable food systems by reducing food waste and loss. Since plastic is now the most effective way to preserve and protect food goods, it is contradictory to expect a long food shelf life to decrease food loss and also decrease plastic waste [3].

Thus, recycling and reusing food packaging is the primary focus of Europe’s Circular Economy agenda, and this problem has also attracted a lot of attention in other sectors. Though packaging eco-designs and recycling technology have noted a significant advancement in the past few years, the recycling rates noted for polymer and plastic-coated packaging materials were seen to be low. For instance, the recovery rate for plastics used in foodservice and packaging industries in the US is roughly 14%. The recycling rate of plastic packages in Europe was thought to be slightly higher, at around 40%, in comparison to ≈80% for cardboard in the continents [4]. A well-managed recycling system is dependent on local recycling capability in addition to sorting and collection infrastructure, which remains inadequate in several countries throughout the world. Mechanical recycling is the most common process that is used for recycling plastic waste in the EU, which includes sorting, shredding, washing, drying, and pelletising the plastic to generate a recyclate material [5].

Pyrolysis (thermal decomposition) has emerged as a promising option to make plastic waste usable for both waste and energy management [6], and to reduce the overall amount of municipal solid waste [7,8]. Pyrolysis has been used for thousands of years to produce charcoal and carbon, and more recently pyrolysis has been used to produce oil, activated carbon, coke, carbon fibre, and methanol [9,10]. Essentially, a distinction is made between fast and slow pyrolysis. Fast pyrolysis is a thermal cracking process that takes place at very high heating rates with very short steam residence times at high pyrolysis temperatures of about 450–1000 °C, and of which the main goal is to reduce secondary reactions to unstable products. This maximises oil yield, which can be increased by up to 75% [11,12]. Slow pyrolysis is a process in which the starting material is slowly heated in the absence of air. Instead of burning, the volatile components of the organic material are partially evaporated, leaving behind the charred product, which makes up a large proportion [13].

In general [14], the main chain structure of polymers is cleaved randomly at weak links of the main chain, at one end of the chain or in labile structures when exposed to heat. Depending on the location of the reaction in the polymer structure and the conditions chosen for the particular system, the resulting radicals follow different reaction pathways, from the depolymerisation of the main chain to inter- or intramolecular transfer reactions. Pyrolysis at high temperatures (=fast pyrolysis) makes most pyrolysis processes uneconomical and dangerous, and can also generate highly toxic gases that must be treated before they can be released into the atmosphere. The advantages of slow pyrolysis over fast pyrolysis are many; in particular, the long duration of slow pyrolysis leads to better heat transfer and, in the case of plastics such as High Density Polyethylene (HDPE) and Polypropylene (PP) [15,16], to a high liquid yield. The limitations of slow pyrolysis include low yields of liquid products and the removal of carbon from the liquid products [17]. In addition, temperatures higher than 500 °C are reported to be required for the pyrolysis of polyethylene and PP [18,19]. However, the direct slow pyrolysis of plastics produces a variety of compounds, including wax, which can lead to pipe blockages [18] and instability during storage [20,21].

A detailed understanding of slow pyrolysis, including the impact of long duration on product distribution and the desired value addition, is still lacking and only a few reports are available [22]. As also suggested in [23], an advanced pyrolysis technology needs to be developed. The advanced pyrolysis technology is based on modifications of conventional pyrolysis processes, which are expected to improve product yield, quality and properties, and reduce pyrolysis temperature and time. However, recent developments in the field of slow pyrolysis show that slow pyrolysis may have an advantage over fast pyrolysis to increase the production of high-value liquid products [22].

Earlier studies showed that nonthermal plasma processing (NPP) can offer an excellent solution to the aforementioned technical issues [24]. Because of its distinctive nonequilibrium reaction environment, the nonthermal plasma (NTP) technique has been used in the pyrolysis of plastics to yield liquid fuels. Additionally, the NTP reactor offers various advantages compared to the catalytic method, including the ability to operate at low temperatures and air pressures, as well as decreased coke formation [25]. The NTP process generates reactive species, and the plasma’s nonequilibrium properties can bypass the thermodynamic limitations noted in chemical reactions [26]. When compared to the traditional hydrodeoxygenation process, NTP upgrading offers additional processing benefits, such as a greater conversion percentage (Y_conv%_) and a higher production of deoxygenated products [27]. Ref. [28] stated that the use of NTP generates a high concentration of species in their excited state, allowing for the occurrence of chemical reactions with high activation energy. Owing to the lower activation energy barrier, NTP can be considered a viable alternative technique to catalyst-based processes to generate species that were undesirable at room temperature. This strategy allowed the researchers to use surrogate compounds to study reaction selectivity and plasma effectiveness. The specialised working conditions of NTP transform polyolefin-derived petrochemical compounds at an atmospheric pressure with low energy usage, and yield good laboratory results. Direct reforming further enhances the gaseous nature of petrochemicals by secreting hydrocarbons into gas streams. However, further study is required to demonstrate the reliability of using the NTP waste polyolefin cracking process with regards to reaction predictability, upscaling, and energy efficiency.

Upgrading applicable models to predict product yields under various functioning conditions is crucial in the reusing and recycling of food polyolefin packages, and has obtained much attention in recent time. At the same time, plasma modelling investigations have improved from a basic charged ideal gas model [29] to multiscale modelling methods, for instance, the complicated fluid and wave plasma models [30]. In one study, the researchers proposed modelling that explained NTP as a sequence of reaction kinetics, focusing on the chemical reactions and their kinetics within the NTP system [31]. This method facilitates researchers to converge on plasma response capacities by modifying plasma modelling into a process to calculate the generation and/or loss of numerous thermochemical reactions yields employing a few reaction rate equations. Conversely, model validation to the experimental data and multidimensional (2D and 3D) visualisation are challenging when non-linear electrochemical parameters were seen to be the key variables that affect the chemical reaction rates. Unfortunately, many of the published studies focus on the phenomenological effects of plasma-assisted valorisation (PAV), while the basic chemical and physical mechanisms of plasma enhancement were not explained. Thus, the researchers have not examined the reaction pathways and determined which reaction factors played a primary role. These issues can be addressed by offering a solution that combines numerical and experimental approaches.

To the best of our knowledge, this was the first report where researchers proposed designing an optimisation model based on four electrical factors (such as power discharge rate ($x_{1}$), discharge interval ($x_{2}$), power frequency ($x_{3}$), and power intensity ($x_{4}$)) that affected the conversion yield (Y_conv%_) of solid plastic packaging materials to liquid fuels. In this study, the researchers carried out a thorough theoretical analysis of model fitness. The complicated structure of plasma chemistry, which comprises many reactions and products, as well as their temporal features, makes modelling and simulation difficult, resulting in computing challenges and expensive simulations. The zero-dimensional (0-D) technique avoids the necessity to model the bulk plasma properties. The 0-D technique is an appealing initial step for evaluating plasma chemistry and determining important reaction rates in NTP without requiring a lot of computational time due to its simplicity [32,33,34,35].

Furthermore, a few primary statistical techniques, like the response surface model (RSM), can be used for data fitting. The RSM technique presents an experimental design which uses mathematical and statistical techniques to develop models to yield a response after including several factors within the system. This data fitting approach can be applied in various domains, including catalysis, material science, and hydrocarbon thermal cracking models [36,37,38,39,40,41,42,43]. To optimise and validate the kinetic model, the researchers used lab-scale or pilot-scale experimental data derived from several substrates in reactors with different topologies and reaction circumstances. The properties of extracted fuel were utilised to determine its quality.

In the past, very few researchers evaluated the progression of the non-catalytic pyrolysis volatiles’ electrochemical process variables that affect nonthermal plasma (NTP) pyrolysis. The objective of this study was to use food packaging plastics as a hydrogen donor to improve the quality of the produced liquid fuels. The application of NTP pyrolysis to polymers was investigated in earlier studies [44]; however, it requires a catalyst, which complicates the process and hinders process optimisation. The data presented in the 2022 Plasma Roadmap [45] indicated that optimisation and scale-up were some of the major technological obstacles that affect chemical feedstock treatment and hydrocarbon production. The data also showed that the development and application of various plasma-science-linked advancements necessitate the optimisation and scale-up of techniques from lab-scale trials to industrial levels, and finally to society level.

## 2. Modelling and Experiments

### 2.1. Model Development

Plasma chemistry processes like excitation, de-excitation, ionisation, and recombination take place on a microsecond (ms) time scale and require several computational tasks. Thus, several plasma models cited in previous studies were zero-dimensional (0-D), resulting in lower time and computation costs [46]. This study combined the 0-D model equations with a multivariable optimisation model, yielding a distinctive hybrid model that incorporates the electrochemical reaction’s Y_conv%_ computations and presents all the results in a 3D mode. This was ascribed to the interface of electrochemical process variables that were employed in the Stat-Ease-360 software module (version 23.1.0).

When the required process parameters’ maximum and minimum points were defined numerically, the simulation became a static study. The model employed a cylindrical dielectric barrier discharge (DBD) plasma reactor with an axial high-voltage (HV) electrode which was encased in the cylindrical copper (Cu) tube as dielectric and a ground electrode on the external circumference of the dielectric. Pyrolysed gas and nitrogen gas (N_2_) fill the space between the inner surface of the dielectric and the axial HV electrode, resulting in NTP. The pyrolysed gas is carried by N_2_.

### 2.2. Model Hybridisation

Figures of Merit

The 0-D Plasma-Perfectly Stirred Reactor (Plasma-PSR) model is used to simulate the kinetics of NTP-energised reactions, which ensures a uniform distribution and good diffusivity of plasma species within the reactor. The equations covered the transportation of neutral and charged species in addition to Poisson’s equation for the electric field. The model’s basic assumptions are listed below:

1. The energy variables and discharge qualities are considered to change solely in the direction that was perpendicular to the electrodes, allowing for a 0-dimensional simulation;
2. The drift/diffusion approximation describes the flux of charged particles;
3. This 0-dimensional model assumes that every component possesses a high diffusivity and diffuses through the discharge zone after their formation, resulting in an even distribution of spatial variables within the plasma reactor;
4. The electron energy distribution function (EEDF) is believed to be Maxwellian, and the electron temperature equation could be solved;
5. It is assumed that the gas temperature is the same as that of the ions and excited neutral species.

The estimated mass of waste-plastic-derived fuel (pre- and post-treatment) allowed for the prediction and analysis of the NTP system’s performance with respect to varying electrical factors. The figures of merit shown below were estimated. The first included the degradation efficiency, XD, which involves the Y_conv%_ of thermally-pyrolysed fuel to superior plasma-treated fuel (PTF):

(1) $$X_{D}=\left(1-\frac{C_{p}}{C_{p0}}\right)\times 100$$

where C_p_ indicates the PTF mass, while C_p0_ refers to the mass of nontreated fuel (NTF).

In the case of 0-order kinetics, the model provides an equation for calculating the degradation efficiency that is independent of the mass transfer coefficient, suggesting that degradation was controlled kinetically:

(2) $$\frac{X_{D,0}}{100}=\frac{A_{S}}{V_{liq}C_{p0}}k_{r,0}t$$

where $A_{S}$ refers to the interfacial area for treatment and V_liq_ denotes the liquid bulk volume, while k_r,0_ was seen to be the intrinsic reaction rate constant for 0-order surface reactions. In the case of hollow electrodes, As was equal to the area of the immersed electrode area and k_r,0_ possesses the units of moles/unit time/electrode area and was 4.7 × 10^7^ mol/m^2^s.

The following conservation equations were used in this study for the 0-D plasma model [33,47,48].

Global mass balance equation:

(3) $$\frac{d}{dt}\left(\rho V\right)=m_{in}-m_{out}=0$$

where ρ and V are mass density and the reactor volume, respectively. m_in_ and m_out_ are the inlet and outlet mass flow rates, individually.

Species conservation equation:

(4) $$\left(\rho V\right)\frac{dY_{k}}{dt}=m_{in}\left(Y_{k,in}-Y_{k}\right)+\omega_{k}VW_{k}$$

where Y_k_ is the mass fraction of product k, W_k_ is the molecular weight of the product k, and $w_{k}$ is the molar rate of chemical reaction per unit volume of the product k.

Electron energy conservation equation:

(5) $$\left(\rho V\right)\left(Y_{e}c_{\nu e}\frac{dT_{e}}{dt}-\frac{R}{W_{e}}T_{e}\frac{dY_{e}}{dt}\right)=m_{in}Y_{e,in}c_{pe}\left(T_{e,in}-T_{e}\right)-Q_{loss}^{elas}-Q_{loss}^{inel}+Q_{source}$$

(6) $$Q_{loss}^{elas}=\frac{3VR\rho_{e}}{W_{e}}(T_{e}-T_{g})\sum_{k=1,k\neq e}^{k_{g}}\frac{W_{e}}{W_{k}}\nu_{ek}$$

(7) $$Q_{loss}^{inel}=V\sum_{r=l}^{I_{er}}∆H_{r}q_{r}$$

where $Q_{loss}^{elas}$ and $Q_{loss}^{inel}$ denote the electron impact energy lost as a result of momentum transfer, elastic, and inelastic collision events. The power of charged material propelled along an electric field and transferred into a plasma by Joule heating is called Q_source_ or the effective input power. In the equation, the electron constant specific heat capacity (c_ve_) and the constant pressure specific heat capacity (c_pe_) are also defined. Y_e_ indicates the mass fraction of an electron.

The density of time-scaled species can be calculated by

(8) $$\frac{dn_{i}}{dt}=\sum_{j}\{(a_{ij}^{\left(2\right)}-a_{ij}^{\left(1\right)})k_{j}\prod_{l}n_{l}^{a_{ij}^{\left(1\right)}}\}$$

where $a_{ij}^{\left(2\right)}$ and $a_{ij}^{\left(1\right)}$ refer to the stoichiometric coefficients of the reaction species i on both sides of the reaction j. n_i_ indicates the species density, while k_j_ denotes the reaction rate coefficient of j. The temperature range was unsuitable since NTP-type plasma thermodynamic data were used. Hence, the enthalpies of electron collision reactions employed within the kinetic model were described as enthalpies of processes at 3000 K with user-defined functions [47].

A simple circuit model was used to determine the interaction strength between the electrical variables with regards to Y_conv%_. The discharge voltage (V_d_) was utilised as a boundary condition for Poisson’s equation, using the ballast resistor and power supply voltage.

(9) $$V=V_{d}+jAR_{b}$$

where j refers to the discharge current that was consistently acquired during the computation, while A denotes the area of every electrode. The voltage pulses were applied to the right electrode, while the left electrode was linked to the ground.

In the case of nonequilibrium plasma, Joule heating transmits electrical energy into total energy of gas mixture, compared to equilibrium plasma, which only transfers electrical energy into apparent enthalpy. This implies that some of the energy generated by Joule heating increases the gas temperature. The heat released during chemical reactions was inherently incorporated in the unsteady term of total energy, with chemical energy transforming to sensible enthalpy. The boundary conditions for charged species vary based on the electric field direction at the electrode [49]. A generic expression was presented by inserting parameter a, which was set to 1 if the electric field was directed towards the electrode, and 0 otherwise.

The 0 potential was established on the left boundary, while the gap voltage (V_gap_) was assigned on the right boundary. V_gap_ is calculated using the applied voltage (V_app_) based on the following equation:

(10) $$\frac{{dV}_{app}}{dt}=\left(1+\frac{{2l}_{d}}{\epsilon_{d}L}\right)\frac{{dV}_{gap}}{dt}-\frac{{2l}_{d}e}{\epsilon_{d}\epsilon_{0}L}\int_{0}^{L}[J_{+}-J_{-}]dx$$

where dielectric constants (ϵ_d_) for quartz and silicone rubber were seen to be 4.8 and 3.2, respectively, for silicone rubber.

The electric potential boundary condition, or Poisson’s equation, was defined as V = 0 on the cathode and V = V_dis_ on the anode. Equation (10) was used to modify the V_dis_ during the simulation. Lastly, gas temperature at x = 0, d was believed to be equivalent to electrode temperature that was set at 350 K for the 2 electrodes.

### 2.3. Optimisation Model

#### 2.3.1. Equations Developed for the Experimental Design

Here, the researchers presented a mathematical model that was used for developing the response function, which includes data assessment procedures, multivariate assessments, and optimal input variables that can be used to generate a response function. In general, the response function listed below could be employed to design a multivariate experimental model:

(11) $$Y=f(x_{1},x_{2},\ldots ,x_{k})$$

where Y—is the response function;

x—is the factor;

k—is the number of factors in equation.

The researchers conducted the experiments with the help of the defined functional expression using the selected factors. In step 1, they selected 4 factors, i.e., the periodicity of $x_{1}$, $x_{2}$, $x_{3}$, and $x_{4}$, to assess the response function. Table 1 presents the designations and levels used in the model. It was noted that the experimental design included 32 runs. The mathematical expression [50] that described the 32-run multifactorial experimental design is presented below:

(12) $$(1+x_{1}^{\left(1\right)})(1+x_{2}^{\left(1\right)})(1+x_{3}^{\left(1\right)})(1+x_{4}^{\left(1\right)}+x_{4}^{\left(2\right)}+x_{4}^{\left(3\right)})\to N=32$$

where $x_{1}^{\left(1\right)}$, $x_{2}^{\left(1\right)}$, $x_{3}^{\left(1\right)}$ and $x_{4}^{\left(1\right)}$ are the linear effects of factors $x_{1}$, $x_{2}$, $x_{3}$ and $x_{4}$;

$x_{4}^{\left(2\right)}$ and $x_{4}^{\left(3\right)}$ are factor’ $x_{4}$ quadratic and cubic effects, respectively;

N is the number of elements in the mathematical model that equals to the runs’ number.

Therefore, the general expression of the statistical regression becomes [40]

(13) $$\begin{array}{ll} Y=b_{0}+b_{1}x_{1}+ & b_{2}x_{2}+b_{3}x_{3}+b_{12}x_{1}x_{2}+b_{13}x_{1}x_{3}+b_{23}x_{2}x_{3}+b_{123}x_{1}x_{2}x_{3}\\ & +b_{4}x_{4}+b_{14}x_{1}x_{4}+b_{24}x_{2}x_{4}+b_{34}x_{3}x_{4}+b_{124}x_{1}x_{2}x_{4}\\ & +b_{134}x_{1}x_{3}x_{4}+b_{234}x_{2}x_{3}x_{4}+b_{1234}x_{1}x_{2}x_{3}x_{4}+b_{44}x_{4}^{\left(2\right)}\\ & +b_{144}x_{1}x_{4}^{\left(2\right)}+b_{244}x_{2}x_{4}^{\left(2\right)}+b_{344}x_{3}x_{4}^{\left(2\right)}+b_{1244}x_{1}x_{2}x_{4}^{\left(2\right)}\\ & +b_{1344}x_{1}x_{3}x_{4}^{\left(2\right)}+b_{2344}x_{2}x_{3}x_{4}^{\left(2\right)}+b_{12344}x_{1}x_{2}x_{3}x_{4}^{\left(2\right)}+b_{444}x_{4}^{\left(3\right)}\\ & +b_{1444}x_{1}x_{4}^{\left(3\right)}+b_{2444}x_{2}x_{4}^{\left(3\right)}+b_{3444}x_{3}x_{4}^{\left(3\right)}+b_{12444}x_{1}x_{2}x_{4}^{\left(3\right)}\\ & +b_{13444}x_{1}x_{3}x_{4}^{\left(3\right)}+b_{23444}x_{2}x_{3}x_{4}^{\left(3\right)}+b_{123444}x_{1}x_{2}x_{3}x_{4}^{\left(3\right)} \end{array}$$

where $b_{0},b_{n},...,b_{k},b_{n,n+1},....,b_{n,k},b_{n,n+1...,k}$ denote the predictor coefficients.

After linearising the equation, the researchers used a statistical evaluation procedure called the central composite design (CCD) to develop lab-scale or pilot-scale experimental design sets. The design is expressed in Equation (13). The real variables involved in the experimental design are evaluated using multiple physical units. Hence, the mathematical expression employed in the design was generated using coded factor values.

(14) $$X_{b}=\frac{X_{h}+X_{l}}{2}$$

(15) $$∆X=\frac{X_{h}-X_{l}}{2}$$

(16) $$X_{\left(+1\right)}=\frac{X_{h}-X_{b}}{∆X}$$

(17) $$X_{\left(-1\right)}=\frac{X_{l}-X_{b}}{∆X}$$

where *X_b_* is the basic level of the specific variable;

*X_l_* and *X_h_* are the physical minimal and maximal values of the specific variable accordingly;

ΔX is the average value of the difference between *X_h_* and *X_l_*.

The details for solving the procedure can found in [40].

#### 2.3.2. Calculating the Regression Coefficients

A mass balance was defined using a stoichiometry matrix that contains factors derived from the system composition. Chemical components (or their stoichiometric correlations) refer to independent system factors, whereas each phase constituent (known as end members) is a linear combination species.

The coefficients for Equation (18) are computed using the below matrix formula:

(18) $$b={\left(X^{T}X\right)}^{-1}X^{T}Y$$

where *b* is a matrix p-dimensional column vector of the coefficients;

*X* and $X^{T}$ are the matrix of variables and their transposed version, respectively;

Y is the matrix of the responses.

X denotes a matrix with rows containing the independent variable data for several observations. Every observation corresponds to column 1 of the matrix. The following columns include data that are associated with specific parameters. Each factor has a distinct column for additional interactions, indicators, changes, etc. The Y values are presented in the n-dimensional column vector Y. The matrices displayed above are described in the following manner:

(19) $$b=\left(\begin{array}{c} b_{0}\\ b_{1}\\ \vdots \\ b_{p-1} \end{array}\right)\;\;\;\;\;\;\;\;\;X=\left(\begin{array}{cccc} 1 & X_{11} & ... & X_{1,p-1}\\ 1 & X_{21} & ... & X_{2,p-2}\\ \vdots & \vdots & \ddots & \vdots \\ 1 & X_{n1} & ... & X_{n,p-1} \end{array}\right)\;\;\;\;\;\;\;\;\;Y=\left(\begin{array}{c} Y_{1}\\ Y_{2}\\ \vdots \\ Y_{n} \end{array}\right)$$

#### 2.3.3. Optimum Value Calculation Method

The output of the response function was optimised with the help of the partial deviation strategy. This technique, however, can fail to generate satisfactory results if the expression of the response function does not include cubic or quadratic presentations of the components, or if a minima x level was acquired. Thus, the surface response strategy may be used to graphically estimate the optimum level of each variable. This method uses equal-level projections to determine the appropriate values for the selected factors. These projections were constructed by dividing the spatial area using parallel/ grid lines. The diagram is composed of 2 unique factors in addition to medium-valued components. Hence, the standard phrase for the response function in each situation was reduced for 2 factors showing maximum or minimal values, indicating only 2 variance levels. The 24 forms of full factorial tests include six $x_{1}x_{2}$, $x_{1}x_{3}$, $x_{1}x_{4}$, $x_{4}x_{3}$, $x_{2}x_{3}$, and $x_{2}x_{4}$ diagrams that were constructed using the Cartesian coordinates.

### 2.4. Model Equations Developed to Calculate the Impact of the Variables via the Response Surface Method (RSM)

The researchers identified the range of the defined response function, i.e., YI, and detailed the steps they used to generate the required expressions. They employed Equation (13) and the coefficient determination strategy described in Equation (19), which includes a statistical review of the mathematical structures, to determine the response functions displayed below. Equation (20) was generated with the help of the coded factors and represents the maximal value of waste plastic Y_conv%_ to liquid fuel by managing the electrical factors such as $x_{1}$, $x_{2}$, and $x_{3}$. Moreover, this equation included $x_{4}$ as the fourth variable. The optimal levels of each factor were forecasted with the help of the following equation:

(20) $$YC=74.55+2.96x_{1}-1.12x_{2}-0.17x_{3}+10.48x_{4}+2.79x_{1}x_{2}+1.25x_{1}x_{3}-1.38x_{1}x_{4}-0.48x_{2}x_{3}+0.88x_{2}x_{4}+1.69x_{3}x_{4}-4.21x_{1}^{\left(2\right)}-0.49x_{2}^{\left(2\right)}+2.67x_{3}^{\left(2\right)}-20.73x_{4}^{\left(2\right)}$$

The researchers also statistically analysed and described Equation (20) in the below sections. The theoretical technique helped eliminate certain irrelevant coefficients from Equation (13). The Results and Discussion Section provides information related to the conformity levels using statistical assessment. To illustrate the response surface graphs, Equation (20) was simplified using real values instead of coded values (Table 2).

Function YI contained the following factors: $x_{1}$ = 2.96, $x_{2}$ = −0.1.12, and $x_{4}$ = 10.48, with component $x_{3}$ after considering several values (−0.17, −0.21, and −0.27). To determine the optimal value for $x_{3}$, the factor values in Equation (10) were replaced successively, while the remaining 3 factors were kept constant using a recommended technique [51,52]. Equation (10) was codified using predictor coefficients.

A new State-ease 360^®^ (USA) software version was used to collect and randomise design points, as well as for statistical modelling and data analysis. The researchers calculated the model fitness tests and compared the results to the values obtained from an analysis of variance (ANOVA) factorial test. The researchers then implemented the 0-D model equations into a new Python platform available in X. The researchers employed polynomial models to measure the impacts of $x_{1}$, $x_{2}$, $x_{3}$, and $x_{4}$ on Y_conv%_, both as individuals and in the multifactorial environment.

### 2.5. Experimental Set-Up

**Figure 1:**
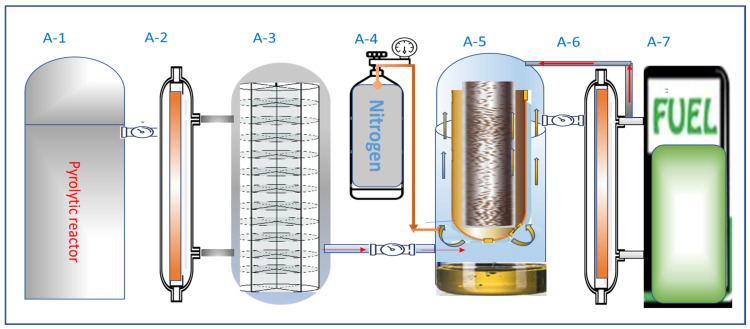
System of the NTP reactor created for the electrochemical treatment of diverse petrochemicals, A-5 inner electrode was connected to a high voltage and variable frequency power supply which was built at Klaipeda University, the Engineering Department [53].

**Figure 2:**
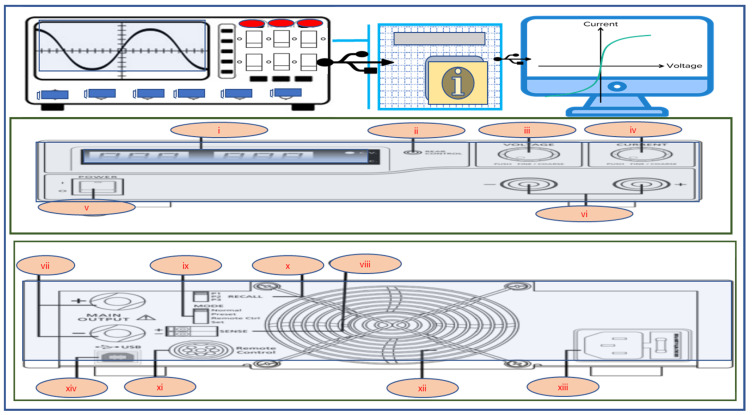
Klaipeda University programmed the laboratory switching mode power supply system, which includes the HCS-3602-USB power supply (i–xiii, display (i), indicator (ii), voltage regulator (iii), current regulator (iv), on–off switch (v), auxiliary current output up to 5 A (vi), high current output (vii), sensor input (viii), control mode switch (ix), recall switch (x), connection to computer (xi), cooling fan (xii), power cord socket (xiii), USB connection (xiv)), including variable frequency voltage source with the flyback transformer (17–50 kHz), high-voltage cables, and using data acquisition equipment for NTP [53].

Here, the researchers employed a cylindrical DBD plasma reactor containing an axial HV electrode that was encased in a cylindrical Cu tube as a dielectric and ground electrode on the dielectric’s external circumference. The researchers filled nitrogen gas (N_2_) in the spaces between the inner surface of the dielectric electrode and the axial HV electrode, resulting in NTP. The system geometry includes the following characteristics (Figure 1 and Figure 2):

The vertical arrangement indicates the tube length of 1.2 m, while the HV electrode is made of a stainless-steel cylinder that has a radius of 2.5 mm and length of 1.18 m. On the other hand, the dielectric Cu tube presented a thickness of 2.5 mm and 25 mm outer radius. The Cu-based ground electrode constituted the outer circumference of the tube, covering 30 cm of its midsection. Nitrogen (N_2_) and copper have a relative permittivity of 1 and 4.2, respectively.

When HV was applied to the inner electrode, it yielded a significant electric field within the gap between all the electrodes. The electric field flux was transferred to the ground electrode from the HV electrode. When an AC voltage was supplied, the direction of the electric field followed the alternating voltage. For all practical applications, plasma was generated at a high electric field to promote the dielectric degradation of gas. In the case of strong electric fields, free electrons gain sufficient energy to implement ionisation reactions. Newly generated electrons migrate towards the boundary surfaces in the opposite direction of the electric field. Equal quantities of ions flow in the same direction like the electric field (as electrons and ions are generated in identical pairs). Consequently, the two boundaries acquire surface charges of an opposite sign.

The polypropylene yoghurt containers that were utilised to produce the discarded polyolefin were acquired from the campus canteen’s trash containers. The containers were washed and ground, and the samples were dried for 48 h. The size of the ground flakes in this study was approximately 10 mm. The experimental setup for solid polyolefin pyrolysis powered by plasma included a 2-stage reactor system, as indicated in Figure 1. Solid polyolefin was initially pyrolysed in the pyrolytic reactor (A-1), and the flow of the gas was directed through a condenser (A-2). After pyrolysis, the liquid was filtered through a filter column (A-3) to eliminate any remaining particle substances. The PP pyrolysis oil showed similar physical properties, like those shown in an earlier study [40]. The researchers used nitrogen gas (A-4) to pass the pyrolysed oil through an NTP reactor (A-5). N_2_ gas acts like a carrier gas and it further prevents the occurrence of negative reactions during the plasma treatment. The treated gas was routed through the next condenser (A-6) that was attached to the fuel collection container. The extra gas was recycled back into A-5 until it was completely condensed.

## 3. Results and Discussion

### 3.1. Verifying the Model

This structure included a few independent variables such as $x_{1}$, $x_{2}$, $x_{3}$, and $x_{4}$. The $x_{2}$ was estimated and measured in units such as ms. The researchers also observed the level of frequency factor that may play an important role in electrochemical polymeric treatment and was expressed in kHz with replication. Table 1 presents the coding and values regarding the factors affecting Y_conv%_ in this study. All independent factors were coded as −1 or +1 to indicate the lower and higher levels of all parameters, respectively.

A maximum of 32 experiments were allowed in CCD (Equation (11)). Table 2 presents a design of all experiments for the 32 runs and their findings. Furthermore, the subsequent sections present the rationale and the discussion of the designed framework, and also describe the changes affecting the variable selections.

### 3.2. Model Explanation and Parametric Effect Analysis via the Integrated Response Surface Method (IRSM)

#### 3.2.1. Multifactor Impact Analysis

The relationship between all variables ($x_{1}$, $x_{2}$, $x_{3}$, and $x_{4}$) and their effects on the response factor (Y_conv%_) must be discovered. The researchers employed the integrated RSM (IRSM) technique to evaluate the influence of all selected parameters on the selected response, identify the optimum values of each variable to get the best response, and calculate the model significance [38]. Equation (20) defines the findings of the RSM-defined quadratic structure used to maximise the Y_conv%_ value.

**Figure 3:**
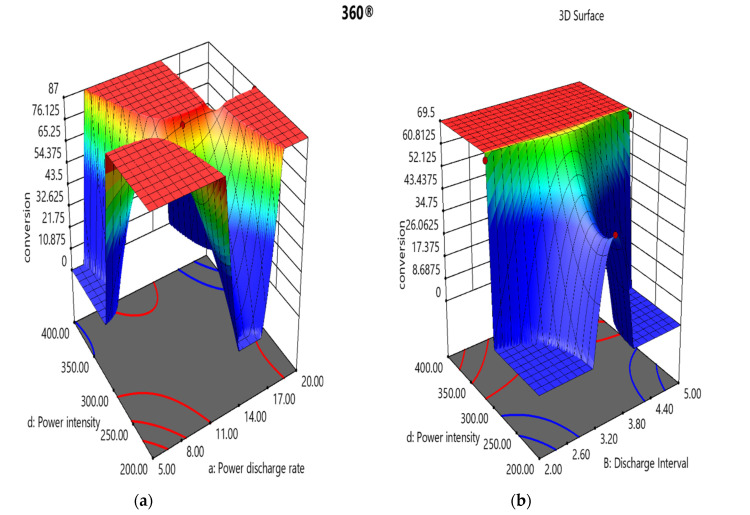

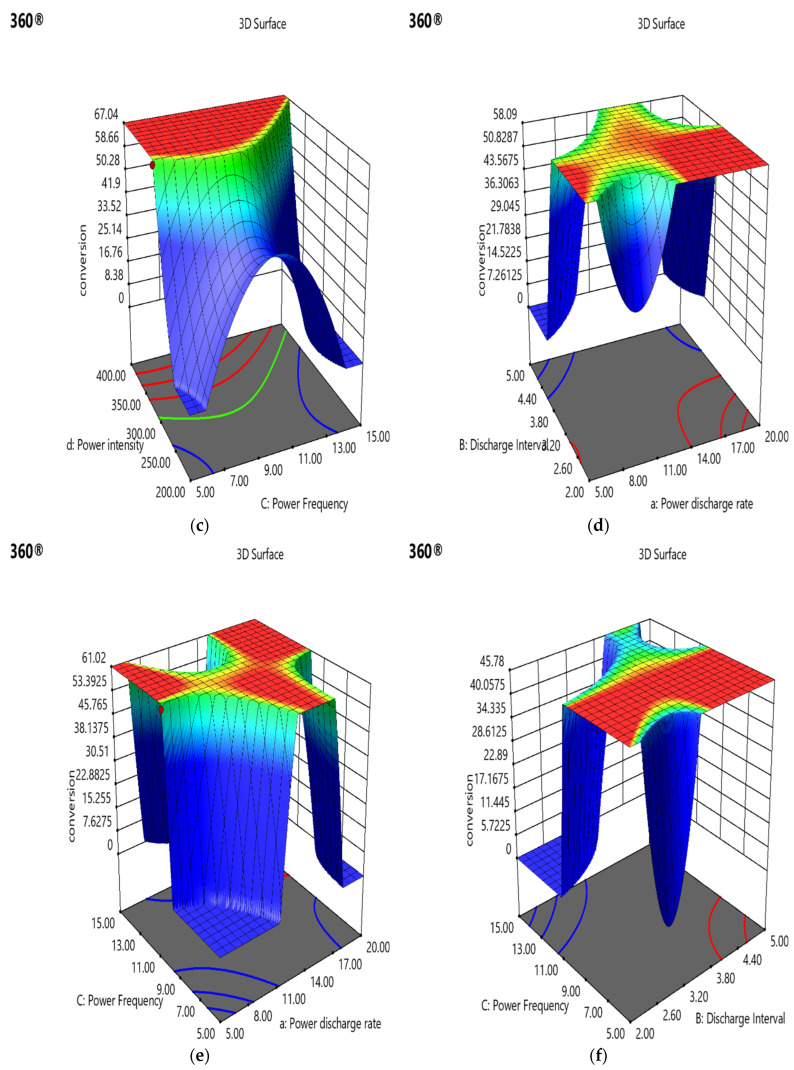
(**a**–**f**) Three-dimensional response plots for NTP parameter interactions (units of the axis labels: conversion in %; power discharge rate in W; discharge interval in ms; power frequency in kHz; power intensity in kV).

The researchers solved Equation (20) to generate response surface plots that offer 3D surface visualisations and contours for different variables (Figure 3). In theory, the impact of two factors might be described by setting the values of other components to the central point of the specified equation and was calculated using the defined process [54]. The results were verified with the help of 3D plots and contour assessments to determine the optimal variable sequence and the minimal Y_conv%_ value on the contour plot surface.

The colours blue and red indicate the lowest and highest Y_conv%_ levels, respectively. This study aimed to determine the maximum potential Y_conv%_; hence, response plots deviating to the red colour represent the probable results. Figure 3a–c exhibits the $x_{4}$-dependent experimental findings. Figure 3a displays the influence of $x_{4}$ and $x_{1}$ on Y_conv%_, whereas $x_{2}$ and $x_{3}$ were kept constant at the centre point of the surface. When $x_{4}$ and $x_{1}$ values reached a particular threshold, the other Y_conv%_ values followed uniformly-distributed ascending trends. Figure 3a displays a 3D response surface map for all the above experiments, emphasising the maximum Y_conv%_ value. The Y_conv%_ showed an ascending trend from 300 kV ($x_{4}$) to 350 kV ($x_{4}$), before plateauing, even though the $x_{4}$ value increased to 400 kV ($x_{4}$). The optimal Y_conv%_ values ranged from 78.71 to 86.42%, after accounting for $x_{1}$ variation. Furthermore, the values showed a significant rise from 12.5 W to 20.0 W. It was seen that when the $x_{1}$ value fluctuated between 15 W and 17.5 W, there was a rapid but equal decrease in Y_conv%_ values, which highlighted the influence of electrical noise from the remaining two variables, $x_{2}$ and $x_{3}$. These outcomes can be used to establish the HV NTP system and determine the tuning ranges for $x_{2}$ and $x_{3}$.

Figure 3b,c exhibits similar trends to the response, wherein a slight increase in the fuel Y_conv%_ value could be attributed to the effect of $x_{2}$ and $x_{3}$ on $x_{1}$. Y_conv%_ increased from 43.05% to 67.04% in both instances, resulting in 69.5%. Figure 3d,e shows the effect of all interactions of $x_{2}$ and $x_{3}$ on $x_{1}$. When the $x_{4}$ value was kept constant, the variations in the Y_conv%_ values indicated that the primary Y_conv%_ values ranged from 43.65% to 45.76%, while the maximal Y_conv%_ ranged between 58.09% and 61.02%. Nevertheless, the interaction between $x_{2}$ and $x_{3}$ exhibited an optimal Y_conv%_ of ≈46%, which maintained the $x_{1}$ and $x_{4}$ values as constant near the surface centre point. The interactions between $x_{2}$ and $x_{3}$ caused significant and unfavourable nonlinearities in Y_conv%_.

However, the interaction between $x_{2}$ and $x_{3}$ resulted in an ideal Y_conv%_ of around 46%, which held the values of $x_{1}$ and $x_{4}$ constant near the centre of the surface. The interactions between $x_{2}$ and $x_{3}$ generated substantial undesired nonlinearities in Y_conv%_. The trend for optimum Y_conv%_ was low, ranging between 7.0 and 11.5 kHz of the $x_{3}$ values. Beyond 11.5 kHz, the Y_conv%_ declined dramatically and continued until 15 kHz. With regards to variations in the $x_{2}$ value, the Y_conv%_ showed the optimal values ranging between 3 ms and 5 ms.

Figure 3a–c represents interactions reliant on the power intensity beside Figure 3d,e, reliant on the power discharge rate, whereas, Figure 3f exhibits the interface between the power frequency and discharge interval impacting on the response Y_conv%_.

#### 3.2.2. One-Factor Effect (OFE) Analysis

The one-factor effect (OFE) plot displays the linear effect that is caused by changing the levels of one variable. It is developed by predicting the responses of one variable at both low (−1) and high (+1) values. OFE graphs play a vital role in factorial designs. The OFE graphs were employed to analyse the effects of the items that were absent in the 3D interaction graph. The graph helped highlight the association between each variable and Y_conv%_ value. The default graphs depict a prediction band on the OFE plot for variables that can influence Y_conv%_. The trends of prediction bands were affected by the design, model, confidence levels, and the inexplicable variation, which were indirectly associated with the results of an ANOVA analysis. If all of the ANOVA indices for the model test showed significant results, these bands were utilised to assess the trends and significant differences to identify the optimal point or points within all predictions. Section 3.3 discusses the findings of the ANOVA test.

**Figure 4:**
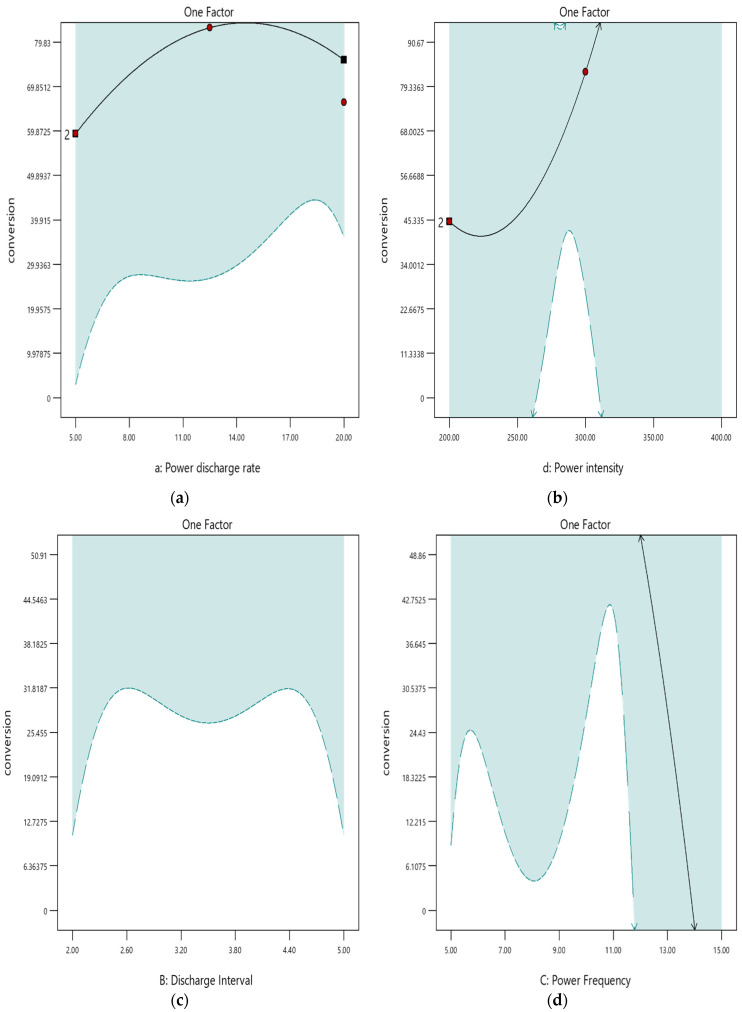
(**a**–**d**) Single-factor impact analysis through the IRSM theory (units of the axis labels: conversion in %; power discharge rate in W; discharge interval in ms; power frequency in kHz; power intensity in kV).

The default view of the OFE shows the average values of significant variables affecting the predictions, which helps with quick comparisons. The predictions differed when the prediction bands did not overlap. The prediction line (Figure 4) is indicated as a black line that originates from the defined forecast. The setting of different factors determined the influence of the factors as they interacted with each other. If a soft response possibility was identified, the OFE plot scan may be somewhat unreliable.

The below-mentioned formula was used for one-factor computation:

(21) $${LSD}_{i}=t_{\left(\frac{\alpha }{2},residual\;df\right)}\frac{\sum_{i\neq j}^{k}\sqrt{(x_{i}-x_{j}){\left(X^{T}V^{-1}X\right)}^{-1}{\left(x_{i}-x_{j}\right)}^{T}}}{k-1}$$

*i*—Point of interest determined by Factors Tool settings and selected treatment on graph;
*j*—Arbitrary reference IDs for each displayed prediction point;
*k*—Total number of displayed prediction points;
*t*—Student’s t critical value;
*α*—alpha risk = 1 − confidence level;
residual df—residual degrees of freedom found on the ANOVA;
$x_{i}$—Expanded point vector for the point of interest or the displayed prediction point;
*X*—The expanded model matrix;
*V*—The variance matrix.

Figure 4a–d shows the apparent effect of a single factor on the response factor (Y_conv%_). The Y_conv%_ values ranged between 59.87–63.58% and 79.83–81.03% because of variations in $x_{1}$ (Figure 4a). Tuning the $x_{4}$ value can show a significant effect within a specified range. The Y_conv%_ values range from 45.33% to 91.03% for the 260–310 kV range (Figure 4b). Optimising the $x_{2}$ and $x_{3}$ factors led to a narrow Y_conv%_ range of 48.86–50.91% (Figure 4c,d).

### 3.3. Analysing Model Accuracy

In the past, several academicians, researchers, and regulators of plastic waste processing depended on analytical accuracy to determine the impact of the outlined structure on optimal Y_conv%_ and its electrochemical process variables. However, solving the polynomial equations in this study allowed the researchers to examine and visualise both the individual and mutual impacts of process characteristics on the Y_conv%_ value, after considering their relevant ranges.

**Figure 5:**
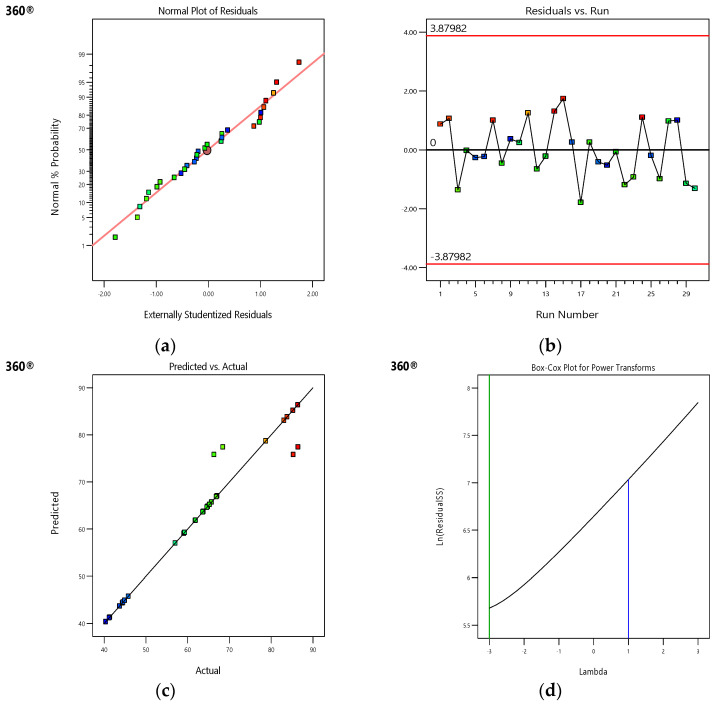
(**a**–**d**) IRSM-based model fitness analysis.

The factors were analysed using several statistical diagnostic techniques and the ANOVA test to evaluate the model’s fitness. First, the range of the factor runs associated with the predicted variables was used to assess the model estimation accuracy. The model indicated 32 runs, and their results could be used for plotting the Y_conv%_ values. The probability and residual plots for Y_conv%_ followed a normal distribution (Figure 5a).

The methods employed in this study confirmed the correlation between expected data and residual-tuned variables ($x_{1}$, $x_{2}$, $x_{3}$, and $x_{4}$), and the findings provided information about distribution normality. The data lined up with the straight line if they exhibited a normal distribution. If the plotted points were close to a straight line, then the data were distributed normally. However, any deviation from the line had the potential to disrupt their normal distribution. Figure 3a compared the data alignment levels to the results obtained using the ANOVA process, where the researchers employed the error terms to determine the deviation level between the observed and model-estimated values. The findings shown in Figure 3a indicate that the values cannot be reproduced since normality excludes additional errors. Residuals refer to errors that are noted during the data assessment process that should be calculated to design statistical models. A systematic assessment of residuals must be conducted to verify the validity of data collection, data processing, and analytical methods. However, the errors were distributed randomly during regression analysis. Figure 5b shows the random distribution of the residual and projected values. This random distribution demonstrated that the data values deviated minimally from the model-estimated values, highlighting the close alignment of the actual and model-estimated values. These elements are explored in the following sections.

The significance of the designed experimental technique and its factors must be evaluated by examining the differences between the point count and ordered set of variables. Outlier measurement is one tool that can help achieve this objective.

Figure 5b depicts the outlier distribution of Y_conv%_ based on the individual variables examined for Y_conv%_. The statistics demonstrate that different configurations provide residuals in the +/−4.00 range, implying a high likelihood of positive agreement between the generated model and response surface. The results indicate that the examined data could possess some errors. On the other hand, variable sequences that exceed the +/−4.00 range were considered insignificant for assessing the data, necessitating additional analysis and recalculation.

The State-Ease 360^®^ software presents a diagnostic Box–Cox plot (Figure 5d). This plot can be used as a reference for determining the optimal λ value for the power law transformation. Figure 5d shows the natural log of the sum of squared residuals (ln residual SS), with λ values ranging from −3 to +3 at the interval of 0.2. The best λ value was seen to correspond to the minimal ln residual value, resulting in a scale that fulfils the equal variance criterion presented in the ANOVA model. This λ value was restricted by the upper and lower 95% confidence ranges. If the 95% confidence interval for λ contained λ = 1 (i.e., no transformation), the software failed to suggest a specific transformation. If not, a particular λ was recommended. However, power law transformation cannot be applied to the responses <0; therefore, a constant (k) was added to each response. The proposed transformation produced the lowest value for the ln residual SS. Additionally, the researchers used the Box–Cox graph transformation (Figure 5d) to identify if data transformation was needed for response modelling. The actual value (λ = 1) ranged between the low and high confidence ranges (−2.89 and 1.34), eliminating the need for model transformation. This indicates the absence of variance and the analytical hypothesis was satisfied.

### 3.4. Perturbation Graph

**Figure 6:**
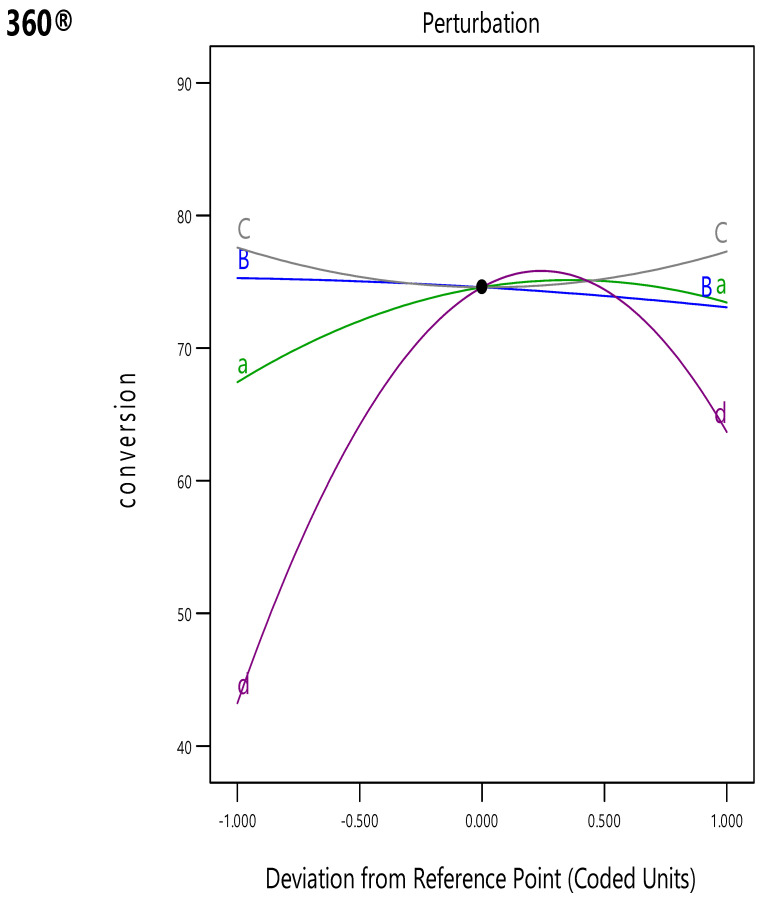
Level of intensity of NTP parameter calculation through the perturbation principle (unit of response, conversion in %).

A perturbation plot is a statistical visualisation approach that can be used for determining the influence of independent components on the response factor, which was necessary to develop an NTP power structure for Y_conv%_. It was based on centre point of the design plot. This plot allows researchers to evaluate the relevance of various variables. Figure 6 presents a perturbation plot for Y_conv%_ response (YIreal) using four inputs, such as $x_{1}$ (A), $x_{2}$ (B), $x_{3}$ (C), and $x_{4}$ (D).

This chart focused on determining the effect of one variable on the displayed response values, where the value of a single variable was changed from its optimal value, while the values of other variables were kept constant.

Figure 6 presents the perturbation plot for the developed model. It shows how each variable affects the Y_conv%_ response during Y_conv%_ optimisation. The figure displays steeper bends for $x_{1}$ (A) and $x_{4}$ (D), indicating that the Y_conv%_ response was more sensitive to the dynamic sequence of all factors that were used for analysing the NTP variables. However, the relatively flat curves for $x_{2}$ (B) and $x_{3}$ (C) indicated that these factors showed a smaller impact on Y_conv%_ sensitivity compared to the 2 other variables. Thus, $x_{2}$, followed by $x_{3}$, displayed a modest influence on the reaction in comparison to $x_{1}$ and $x_{4}$. The variables that were used in the perturbation plot were ranked in the following order: $x_{4}$ > $x_{1}$ > $x_{2}$ > $x_{3}$.

### 3.5. Characterisation of Fuel by Calorific Value

The calorific value is defined as the amount of energy that is generated when a unit mass of fuel completely burns in air. The Parr Instrument^®^ 6400 Bomb Calorimeter (Moline, IL, USA) was employed to calculate the calorific value of plastic fuel. After examining the samples used in this study, the researchers calculated the calorific value of liquid hydrocarbon after NTP treatment which was 43,570.5 J/g. This value was similar to the calorific values displayed by traditional fuels such as kerosene, diesel, and petrol [55]. In the past, a few researchers [56] found that HDPE, Low Density Polyethylene (LDPE), and PP exhibited calorific values close to 40 MJ/kg, which indicates that it can be used as fuel. On the other hand, pyrolytic oil made from Polyethylene Terephthalate (PET) and Polyvinyl chloride (PVC) displays a poor calorific value (<30 MJ/kg). Furthermore, in one study [57], the researchers converted municipal plastic trash to plastic fuel, which displayed a calorific value of 39.72 MJ/kg that was lower compared to the calorific value in this study.

### 3.6. Sensitivity Analysis

**Figure 7:**
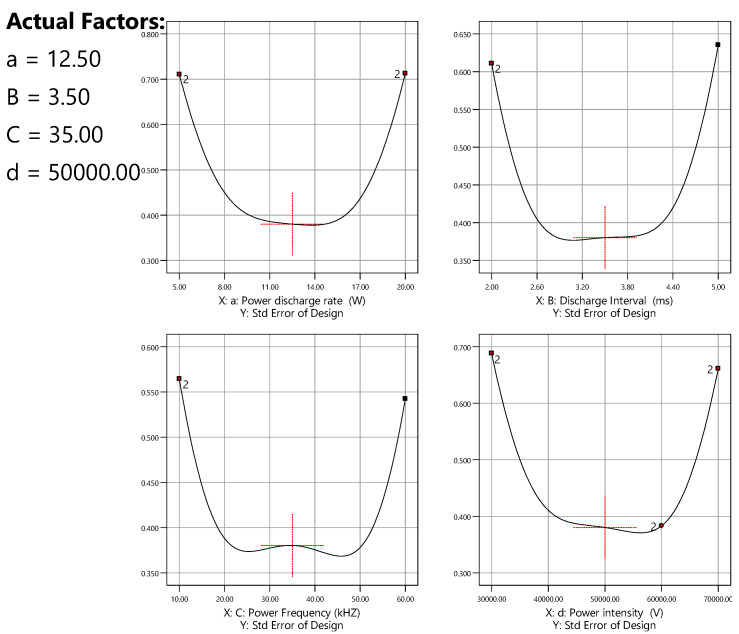
Standard error outlines a four-factor RSM optimisation design on 32 NTP process parameters (units of the factors: conversion-%; power discharge rate in W; discharge interval in ms; power frequency in kHz; power intensity in kV) Theoretically, in standard error calculation under RSM, the error ranged used to fix between 0 to 2 (the number at the begin and end of curves in the diagrams), where the value towards zero is always expected. In this study, all the values remain between 0.300–0.400, which is highly expected.

Besides several parametric impact analysis methods under of optimal design, the ‘sensitivity analysis through the standard error approach’ for multifactorial experimental designing is primarily used in industrial trialling. Adjacent to mired down in the purely mathematical details for these multifactorial criteria and the multitude of algorithms for employing them, it is advisable to focus on in what way they diverge in real application to designed RSM experiments to fit quadratic polynomials, such as the equations shown above. Comparing these plots (Figure 7) side-by-side delivers a message of the comparative feature of predicted response at different spots bridging the experimental region (presented here pertaining to coded values from the centre). A larger replication of points by the responses is required for RSM since it reduces the standard error of calculation at the centre—the point of highest interest—and also delivers a profile for a wide-ranging scope in the centre of the investigational section.

The graphs in Figure 7 provide scales on the standard error minimums, averages, and maximums. For RSM functions, the optimum point delivers an anticipated adjustment of being lower, which is better within an acceptable range of error [58].

## 4. Conclusions

Here, the researchers used numerical and experimental techniques to study the effect of $x_{1}$, $x_{2}$, $x_{3}$, and $x_{4}$ on the Y_conv%_ of plastic food packaging wastes to liquid hydrocarbon. The NTP kinetic parameters combined the simulation equations presented by the 0-D model and CCD-based RSM. A hybrid model that combined the electrochemical plasma procedure, and an RSM framework-based time-fixed 0-D model was designed to describe the evolution of defined variables during plastic degradation. The 0-D RSM model could accurately predict the experimental design, and the generated model can be used to understand the experimental results. The researchers employed ANOVA analysis to examine the relevance and appropriateness of the regression models and understand the relative significance of all the factors used in the plasma treatment procedure. The influence of independent processing variables and their interactions on the reaction performance was completely investigated using 3D response surfaces. The 3D figures reveal that the relationship between $x_{1}$ and $x_{4}$ had a major effect on all responses, but other interactions showed varying influences on the responses of plasma process variables on Y_conv%_. The process optimisation technique determined the optimal process operating parameters ($x_{4}$ = 300 kV, $x_{1}$ = 15.0 W, $x_{2}$ = 3 ms, and $x_{3}$ = 15 kHz). The above-mentioned conditions were confirmed by reproducible experimental data under theoretical optimal factors, which yielded an Y_conv%_ of 86.42%. The perturbation findings demonstrated that $x_{4}$ was a very significant variable that affected Y_conv%_, where $x_{1}$ played a supporting role in determining the efficiency of the plasma parameter arrangement for experiment design. The predicted values of the optimised model for the 2 main variables resulted in Y_conv%_ values of 79.83% and 90.67%, indicating high reaction performance, respectively. On the other hand, the remaining process factors ($x_{3}$ and $x_{2}$) showed low optimal values for the response factor, ranging from 48.84% to 50.91%. It should be emphasised that by preserving the other three variables at their optimised values, this study could reflect the results for OFE on Y_conv%_. Furthermore, it was observed that the converted hydrocarbon’s physical characteristics and fuel attributes, such as its density of 0.8654 kg/l and calorific value of 38.53 MJ/kg, were comparable to those of traditional diesel fuel.

The main goal for the future is to develop totally predictive and reliable physics- or chemistry-based computer models that could be used for designing and optimising the device. This needs thorough investigation in model selection, development, and validation. Some of the physical and technical problems observed in the adoption of the plasma devices and their optimisation included the construction of small and miniature devices for plasma treatment and determining the different plasma and target factors during treatment. The purpose was to dynamically adapt plasma properties to the objective’s actual (short-term) attributes while taking into account the specific product conversion, which is dependent on both target and plasma monitoring.

## Figures and Tables

**Table 1:** Coded alignment of the designated factors.

Factor	Name	Units	Change	Type	Subtype	Minimum	Maximum	Coded Low	Coded High
a	Power discharge rate	W	Hard	Numeric	Continuous	5.00	20.00	−1 ↔ 5.00	+1 ↔ 20.00
B	Discharge Interval	ms	Easy	Numeric	Continuous	2.00	5.00	−1 ↔ 2.00	+1 ↔ 5.00
C	Power Frequency	kHz	Easy	Numeric	Continuous	5.00	15.00	−1 ↔ 5.00	+1 ↔ 15.00
d	Power intensity	kV	Hard	Numeric	Continuous	200.00	400.00	−1 ↔ 200.00	+1 ↔ 400.00

**Table 2:** Model-derived CCD experimental design.

	Factor 1	Factor 2	Factor 3	Factor 4	Response 1
Run	a: Power Discharge Rate	B: Discharge Interval	C: Power Frequency	d: Power Intensity	Conversion
	W	ms	kHz	kV	%
1	12.5	2	5	300	83.81
2	12.5	3.5	10	300	83.07
3	15	3.5	15	300	68.43
4	15	3.5	10	400	63.63
5	20	5	15	200	44.51
6	20	2	5	200	43.69
7	15	3.5	5	300	85.18
8	15	2	15	400	64.81
9	5	2	15	200	41.21
10	5	5	5	400	59.09
11	5	2	10	300	78.71
12	20	5	15	400	64.61
13	5	2	5	400	61.79
14	15	5	15	300	86.38
15	20	3.5	10	300	85.29
16	12.5	3.5	10	200	44.91
17	12.5	3.5	5	300	65.21
18	20	2	15	400	65.71
19	20	5	5	200	45.78
20	5	2	5	200	41.34
21	20	5	5	400	63.73
22	12.5	2	10	300	67.04
23	12.5	5	10	300	66.87
24	15	3.5	15	300	86.42
25	20	2	15	200	44.34
26	20	3.5	10	300	66.31
27	5	5	15	400	61.87
28	5	5	5	200	40.36
29	5	3.5	10	300	59.28
30	5	5	15	300	57.01
31	15	2	10	200	67.07
32	12.5	2	10	400	68.9

## Data Availability

All data generated or analysed during this study are included in this published article.
